# Structure and Kinetic Stability of the p63 Tetramerization Domain

**DOI:** 10.1016/j.jmb.2011.11.007

**Published:** 2012-01-20

**Authors:** Eviatar Natan, Andreas C. Joerger

**Affiliations:** Medical Research Council Laboratory of Molecular Biology, Hills Road, Cambridge CB2 0QH, UK

**Keywords:** DBD, DNA-binding domain, nESI-MS, nanoflow electrospray ionization mass spectrometry, PDB, Protein Data Bank, SeMet, selenomethionine, RMSD, root-mean-square deviation, SAM, sterile α-motif, TAD, transactivation domain, p53 family, tetramer, kinetic stability, X-ray crystallography, nanoflow electrospray mass spectrometry

## Abstract

The p53 family of transcription factors—comprising p53, p63 and p73—plays an important role in tumor prevention and development. Essential to their function is the formation of tetramers, allowing cooperative binding to their DNA response elements. We solved crystal structures of the human p63 tetramerization domain, showing that p63 forms a dimer of dimers with *D*_2_ symmetry composed of highly intertwined monomers. The primary dimers are formed via an intramolecular β-sheet and hydrophobic helix packing (H1), a hallmark of all p53 family members. Like p73, but unlike p53, p63 requires a second helix (H2) to stabilize the architecture of the tetramer. In order to investigate the impact of structural differences on tetramer stability, we measured the subunit exchange reaction of p53 family homotetramers by nanoflow electrospray mass spectrometry. There were differences in both the kinetics and the pattern of the exchange reaction, with the p53 and p63 tetramers exhibiting much faster exchange kinetics than p73. The structural similarity between p63 and p73 rationalizes previous observations that p63 and p73 form mixed tetramers, and the kinetic data reveal the dissociation of the p73 homotetramers as the rate-limiting step for heterotetramer formation. Differential stability of the tetramers may play an important role in the cross talk between different isoforms and regulation of p53, p63 and p73 function in the cell cycle.

## Graphical Abstract

**Figure:**
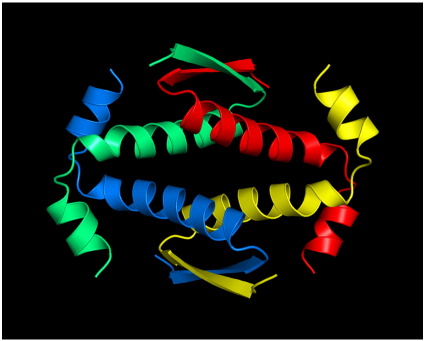

**Highlights:**

► The transcription factors p53, p63 and p73 are active as tetramers. ► We solved crystal structures of the p63 tetramerization domain. ► Like p73, but unlike p53, p63 requires a second helix to stabilize the tetramer. ► The p73 tetramerization domain has a higher kinetic stability than p63 and p53. ► Differential stability of the tetramers may be of functional importance.

## Introduction

**Fig. 1:**
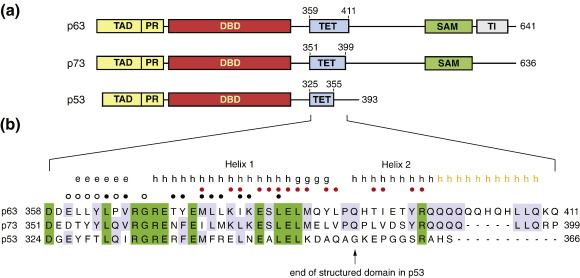
Structural organization of human p53, p63 and p73. (a) Schematic comparison of the domain structure of full-length p53, p63 and p73, showing the N-terminal TAD, proline-rich region (PR), DBD, tetramerization domain (TET), SAM domain (p63 and p73) and transactivation inhibitory domain (TI). See the text for further details. (b) Alignment of p53 family tetramerization domain sequences. The C-terminal amino acids shown for each family member correspond to the 3′-end of exon 10 encoding the tetramerization domain.^5^ Residues conserved in all three family members are highlighted in green, whereas residues conserved in only two members are highlighted in light blue. Secondary-structure elements in the crystal structure of p63(359–402) are indicated by black letters: e (β-strand), h (α-helix) and g (3_10_-helix). Orange letters show the results from consensus secondary-structure prediction for the residues not resolved in the crystal structure using the NPS@ protein sequence analysis Web server.^6^ p63 residues forming interdimer contacts are marked with red circles, whereas black circles denote residues involved in intradimer contacts. Residues with black open circles contribute to this interface primarily via their main-chain atoms.

The transcription factors p53, p63 and p73 play an important role in cancer prevention, development and longevity.^1–4^ The three proteins share a similar domain organization (Fig. 1a), being composed of structured DNA-binding domain (DBD) and tetramerization domain flanked by intrinsically disordered regions.^2,7,8^ The phylogenetically more ancient p63 and p73 proteins have an extended C-terminal region that is absent in p53. This region contains a structured sterile α-motif (SAM) domain^9–11^ and, in the case of p63, an additional inhibitory domain at the extreme C terminus that negatively regulates the transcriptional function of p63.^12^ The proteins are active as tetramers, allowing cooperative binding of four DBDs to target promoter sites.^13^ Tetramers are formed via a short tetramerization domain that follows the DBD, with a short flexible linker region separating the two domains. Solution and crystal structures of the tetramerization domain have been solved for p53 and p73,^14–19^ but not for p63. Both p53 and p73 form dimers of dimers with *D*_2_ symmetry. The p73 tetramerization domain contains an additional C-terminal helix that is important for stabilizing the tetramer.^17,18^ The three family members can interact with each other, although two fundamentally different molecular mechanisms for interaction have to be distinguished: formation of mixed tetramers and co-aggregation. The oligomerization domains of p63 and p73 can form mixed tetramers, suggesting functional cross talk, whereas p53 does not form heterotetramers with its family members.^17,18,20^ Some p53 cancer mutants possess oncogenic gain of function via interaction with p63 and p73 despite the tetramerization domains not interacting with each other. A recent study has shown that this effect is caused by an aggregation-prone sequence within the hydrophobic core of the DBD, which is conserved in the p53 family.^21^ This region is exposed in conformationally unstable p53 cancer mutants, triggering co-aggregation with wild-type p53, as well as p63 and p73. A large number of isoforms of p53, p63 and p73 can be expressed as a result of alternative splicing of C-terminal exons or the use of two alternative promoters.^5^ For p63 and p73, the latter results in isoforms with either an intact transactivation domain (TAD), essential for the transactivation of target genes (TAp63 and TAp73), or dominant-negative isoforms lacking the TAD (ΔNp63 and ΔNp73). Interplay between the different isoforms via formation of mixed tetramers is thought to be an important mechanism in controlling the overall function of p53 family members.^8^ A recent study has shown that p63 function in mouse oocytes is regulated via the oligomerization state of p63.^22^ In its latent state, p63 forms dimers with reduced transcriptional activity through an intramolecular inhibition of tetramer formation involving the N and C termini interacting with the tetramerization domain.

Here, we report crystal structures of different p63 tetramerization domain variants, showing not only marked similarity to p73 but also small differences that may be important for regulating the function of p63 *in vivo*. In addition, we measured the kinetics of subunit exchange for different p53 family members using nanoflow electrospray ionization mass spectrometry (nESI-MS). These data show that the p73 tetramer is kinetically the most stable of the three p53 family members, which has potential implications for the functional cross talk of different isoforms and formation of p63/p73 heterotetramers.

## Results

### Structure of the human p63 tetramerization domain

**Fig. 2:**
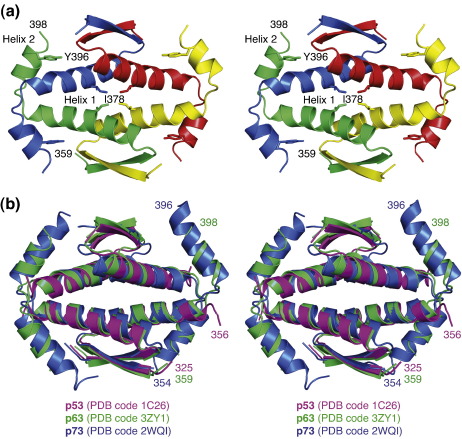
Stereo view of the p63 tetramerization domain structure. (a) Structure of the p63(359–402) tetramer shown as cartoon representation. Individual subunits are shown in different colors. Selected side chains at the tetramer interface are shown as stick models and labeled. (b) Superposition of the tetramerization domains of p63, p73 (PDB code 2WQI)^17^ and p53 (PDB code 1C26).^14^ The tetramers are superimposed based on one of the two primary dimers, for example, the yellow and green chains in (a).

Initial crystallization trials with a human p63 tetramerization domain variant comprising residues 356–411, p63(356–411), resulted only in poorly diffracting crystals. We then systematically truncated the C-terminal region, which significantly improved the crystal quality, and we were able to solve the structure of p63(359–402). This tetramerization domain variant comprises the equivalent of the C-terminal residues forming key interactions in the crystal structure of the p73 homolog.^17^ In addition, we also solved the structure of a variant lacking the C-terminal helix altogether, that is, containing only the structural elements of the canonical p53 tetramerization domain motif (Fig. 1b). Both structures were solved using selenomethionine (SeMet)-substituted proteins and multiwavelength anomalous dispersion phasing. The crystal structure of the long variant was solved at a resolution of 2.15 Å. The crystals belonged to space group *P*422 and contained one monomer in the asymmetric unit. Like its family members, p63 forms dimers of dimers with *D*_2_ symmetry (Fig. 2). The overall structure of the p63 tetramerization domain is very similar to that of p73 (2WQI; an RMSD of 1.5 Å over the aligned C^α^ atoms with 55% sequence identity). The monomers consist of a β-strand followed by two helices, H1 and H2, the latter being absent in the p53 homolog. They adopt a z-shaped double-hairpin conformation with virtually no intramolecular contacts between the different structural elements. Two such monomers dimerize via intermolecular antiparallel β-sheet and antiparallel packing of the H1 helices (e.g., the green and yellow chains in Fig. 2a). Important hydrophobic contacts are made by Leu364, Val366, Tyr372, Met374, Leu375, Ile378 and Leu382. The total surface area buried within this dimer is 2120 A^2^.

**Fig. 3:**
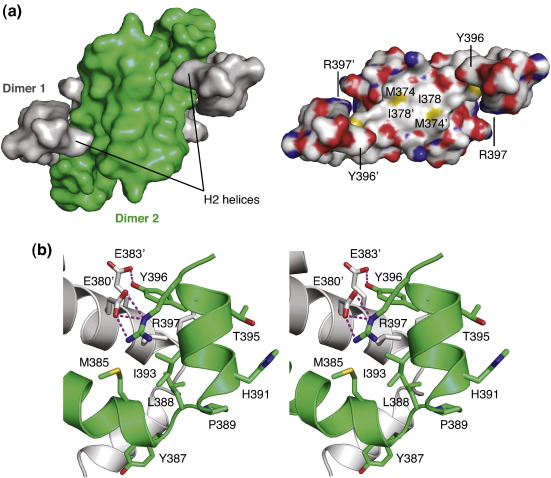
p63 dimer–dimer interface. (a) Molecular surface of the p63 tetramer (left). The view is perpendicular to the β-sheets, showing the C-terminal helices with Tyr396 clasping the neighboring dimer. The two primary dimers are shown in gray and green, respectively. Removal of the green dimer reveals the contact area of the central hydrophobic dimer–dimer interface (right). Selected contact residues in the H1–H1 interface (Met374 and Ile378) and the H2 helix (Tyr396 and Arg397) are labeled. Atom color code: oxygen, red; nitrogen, blue; carbon, gray; sulfur, yellow. (b) Stereo view of the H1/H2 linker region and H2-mediated interactions with the adjacent primary dimer. Polar interactions are highlighted with magenta broken lines.

Tetramers are formed via largely hydrophobic H1–H1 interactions of two primary dimers and H2-mediated contacts (Fig. 3). The H2 helices from one primary dimer reach across and clasp the adjacent primary dimer, with the two primary dimers packing in approximately orthogonal fashion via their H1 helices (Figs. 2 and 3a). Key interacting residues are conserved in p63 and p73, but there are some notable variations, resulting in differences in surface complementarity. At the center of the tetrameric p63 interface, for example, the Ile378 side chains from each subunit contact each other, whereas the corresponding residues in p53 and p73 are leucines (Leu344 and Leu371, respectively). Helix H2, which is essential for the stability of the tetramer, forms both hydrophobic and polar intersubunit contacts: the latter comprise a hydrogen bond between Tyr396 and Glu383′ and a salt bridge between Arg397 and Glu380′ (Fig. 3b). In addition to formation of an intermolecular hydrogen bond, Tyr396 stabilizes the tetramer interface through hydrophobic interactions with Leu384′ and Tyr387′. Tyr396, Arg397 and the interacting glutamates on the H1 helix are conserved in p63 and p73. Residues in the sharp H1/H2 turn and the N-terminal region of H2 are less conserved (see Fig. 1), but the resulting interface with the adjacent subunit is similar in nature and characterized by hydrophobic interactions. Interestingly, the first turn of helix H2 differs in the two orthologs. p63 has a histidine (His391) instead of the proline found in p73, indicating a lower structural rigidity of this region in p63.

The last four residues of the construct used for crystallization, Gln399 to Gln402, were not resolved in the crystal structure, indicating a high degree of flexibility. A comparable conformational flexibility of C-terminal H2 residues was also observed in one of the two crystal forms reported for the p73 tetramerization domain [Protein Data Bank (PDB) code 2WTT]. Based on secondary-structure prediction, helix H2 potentially extends up to residue 409 (see Fig. 1) and would thus significantly protrude beyond the compact core of the tetramer. This region is significantly longer in p63 than in p73 because of a five-residue insertion within the glutamine-rich C-terminal region. The high conformational flexibility of the C-terminal region of H2 would explain the failure to obtain crystals that diffracted well for the longer p63 tetramerization domain constructs.

### Structure of the p63 tetramerization domain upon deletion of the H2 helix

**Fig. 4:**
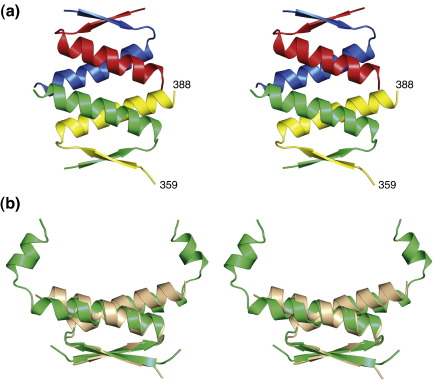
p63 tetramerization domain structure upon deletion of the second helix. (a) Overall structure of the tetramer observed in the asymmetric unit of the crystal structure of p63(359–388). Contrary to the orthogonal packing of primary dimers in the full-length protein, the tetramer is formed via antiparallel packing of H1 helices from adjacent dimers. (b) Superposition of the primary dimers in the structures of p63(359–402) with H2 helix (green) and p63(359–388) lacking this helix (light brown).

Deletion of the H2 helix has been shown to substantially destabilize the p63 and p73 tetramers, and much higher protein concentrations were needed to shift the equilibrium toward tetrameric species.^17,18^ We solved the crystal structure of such a truncated variant of p63, p63(359–388), at a resolution of 1.9 Å. Interestingly, this crystal structure, solved in the space group *P*1, contained one tetramer in the asymmetric unit but with fundamentally different packing characteristics than those observed for the longer variant (Fig. 4). The primary dimers are formed as observed for the longer variant, that is, by formation of an intermolecular β-sheet and antiparallel packing of the H1 helices. These primary dimers, however, pack in an antiparallel fashion to form tetramers, which is in stark contrast to the orthogonal packing observed in the full-length tetramers of p53, p63 and p73. In addition, there are small differences in the structure of the primary dimers, most notably, a difference in packing angle and conformation of the H1 helix. This helix adopts an α-helical conformation throughout, whereas the packing interactions in the full-length variant cause a distortion at the C-terminal end of the helix and a transition to a 3_10_-helical conformation (see Fig. 4b). Key hydrophobic interactions at the center of the primary dimer interface are conserved. Similar shifts in H1 packing angles and conformation have been observed for p73 upon truncation of the C-terminal helix.^17^ In the case of the p73 crystal structure, both tetramers and hexamers were found in the asymmetric unit. As observed for the p63 structure, these higher-order oligomers are formed by antiparallel packing of primary dimer building blocks, in contrast to the approximately orthogonal packing of the primary dimers in the full-length tetramers. Hence, loss of the second helix in p63 and p73 not only weakens the tetramer but also fundamentally alters the overall orientation of the H1 helix packing, revealing a high degree of fluidity of this interface in the absence of the H2 helix.

### Kinetic stability of the p63 tetramerization domain compared to p53 and p73

**Fig. 5:**
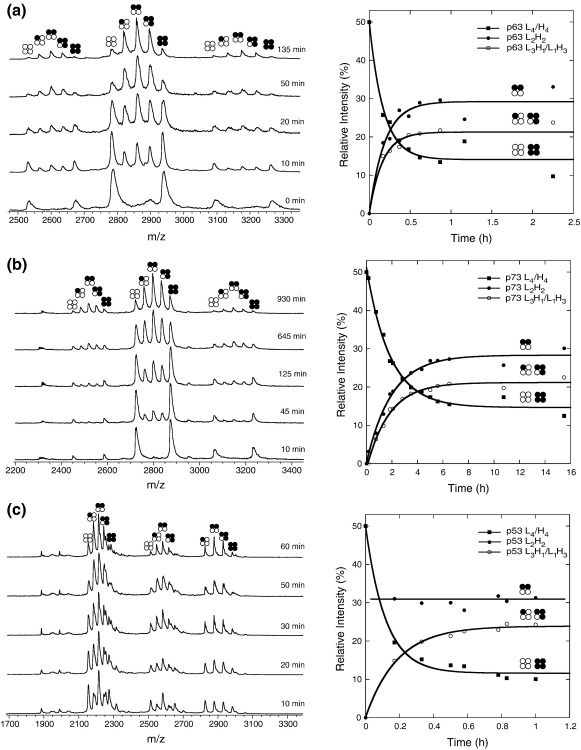
Kinetics of subunit exchange of p53 family tetramerization domains. The exchange of p63 (a), p73 (b) and p53 (c) tetramerization domains at 37 °C was monitored by nESI-MS after mixing ^12^C–^14^N-labeled variants (L; black open circles) and ^13^C–^15^N-labeled variants (H; black filled circles) of each family member. Selected mass spectrometry spectra at different time points are shown (left). The relative intensities of the species were calculated from the spectra and plotted against time (right), showing differences in exchange kinetics and patterns when comparing the different family members. We plotted the averages of the two homotetramers and the two 3:1 complexes (L_3_H_1_/L_1_H_3_).

We measured the kinetic stability of p53, p63 and p73 tetramerization domains by nESI-MS. Equal amounts of unlabeled protein (^12^C–^14^N = “L”) and uniformly labeled protein (^13^C–^15^N = “H”) were mixed, and the distribution of the various tetrameric species at different time points after mixing was monitored by nESI-MS. The measurements were performed at 37 °C using the full-length tetramerization domain variants p63(356–411) and p73(351–399) that had previously been used to measure the formation of mixed tetramers.^17^ The initial concentration of the homotetramers was in the low micromolar range so that the starting species were mainly tetrameric, given that the monomer–dimer and dimer–tetramer equilibrium constants for the different p53 family members are in the nanomolar range.^13,23^ Immediately after mixing, only homotetrameric species were observed, containing either “light” (L_4_) or “heavy” subunits (H_4_). In the case of p63, significant populations of mixed species with L_2_H_2_, L_3_H_1_ and L_1_H_3_ stoichiometries were already observed after 10 min of incubation. After about 50 min, equilibrium was reached. The ratio of L_4_, L_3_H_1_, L_2_H_2_, L_1_H_3_ and H_4_ was 1:4:6:4:1, as expected for a statistical distribution (Fig. 5a). Kinetic modeling showed that the tetramers dissociate with a half-life, *t*_1/2_, of about 7 min (Table 1). For the p73 tetramerization domain, the subunit exchange proceeded much more slowly (Fig. 5b). The p73 tetramers had a 10× longer half-life than the p63 tetramers, and equilibrium was only reached after about 10 h. As with p63, the ratio at equilibrium was consistent with a statistical distribution of subunits.

The p53 tetramer had the shortest half-life of the three family members, with a *t*_1/2_ of about 5 min (Table 1). There was a notable qualitative difference in the subunit exchange process for p53 (Fig. 5c). The 2:2 complexes reached equilibrium almost instantly, whereas the 3:1 complexes were formed much more slowly, in contrast to p63 and p73, for which the complexes reached equilibrium at a comparable rate. These data indicate that intradimer and interdimer interfaces are of a comparable strength in p63 and p73, respectively, but differ significantly in p53 where the dimer interface is much more stable than the tetramer interface. The kinetic stabilities of the isolated tetramerization domains measured by nESI-MS parallel the differential dissociation constants of full-length p53, ΔNp63β and ΔNp73β determined by analytical ultracentrifugation,^13^ showing that the p73 tetramer is the most stable of the three orthologs.

## Discussion

We have solved the crystal structure of the p63 tetramerization domain, thereby completing the picture of the structural evolution of this domain within the human p53 family. The overall structure of the p63 tetramerization domain is very similar to that of the p73 domain but distinct from that of p53 in that it contains an additional helix while retaining the overall symmetry and basic architecture of the tetramer. A common feature of the three family members is the formation of dimers of dimers with *D*_2_ symmetry and approximately orthogonal packing of the dimers. The structural data are consistent with earlier biophysical studies on this domain, showing that the additional C-terminal helix, H2, is essential for stabilizing the tetramer.^17,18^ The H2 helices from one primary dimer reach across the adjacent dimer, thus stabilizing the tetramer. The fact that this helix is found only in the more ancestral and phylogenetically more closely related members, p63 and p73, reflects an evolutionary pathway toward smaller building blocks in p53 that do not interact with their more ancestral family members.^17^

The overall architecture of the p63 and p73 tetramers suggests a zip-like dissociation mechanism by which H2-mediated interactions have to be broken first before the tetramer can dissociate into dimers and monomers. Despite their similar architecture, there are subtle structural differences affecting the stability of the tetramers that may play an important role in the regulation of p63 and p73 transcriptional activities *in vivo*. Our mass spectrometry data on the kinetics of subunit exchange of the different p53 family members show that the p63 tetramer has a significantly lower kinetic stability than the p73 homolog, and accordingly, the p63 tetramers dissociate more rapidly. It will be interesting to see whether this is due to specific variations within the central hydrophobic tetramer interface or the result of a higher intrinsic flexibility of the H2 helix in p63. While the central polar interaction network via the H2 helix is conserved, there are several amino acid variations in the hinge region between helices H1 and H2 and the N-terminal half of H2 (Fig. 1b). One of the two prolines at the N-terminal end of the H2 helix in p73 (Pro384), for example, is replaced by a histidine in p63 (His391), which may affect the conformational flexibility of this helix. Interestingly, a recent study has shown that the activity of TAp63α in mouse oocytes is regulated via its oligomerization state.^22^ In the latent state, the N and C termini of p63 interact with the oligomerization domain, stabilizing a dimeric transcriptionally less active form of p63 and inhibiting tetramer formation. This inhibition is relieved upon phosphorylation, and active tetramers are formed. Chemical shift mapping by NMR suggests that the interaction sites of the H2 helix and the N terminus overlap.^22^ Hence, increased conformational flexibility of helix H2 may be necessary for fine-tuning the regulation mechanism of p63 in mouse oocytes. Given the structural similarity between the p63 and p73 oligomerization domains, similar activity switches via modulation of the oligomerization state may also be involved in regulating p73 function.

We and others have previously shown that p63 and p73 form mixed tetramers, whereas the p53 tetramerization domain does not form heterotetramers with its family members.^17,18^ On the basis of the similarity of the p63 and p73 structures and conservation/homology of key interacting residues, this observation is now easy to rationalize, as the subunits can replace each other without perturbation of the overall architecture of the tetramer. Intriguingly, the 2:2 complex formed by association of two primary dimers of p63 and p73 is thermodynamically more stable than the homotetramers.^18^ Thus, the asymmetric tetramer interface in the mixed tetramer is more stable than the symmetric interfaces of the parent tetramers as a result of improved packing interactions. *In vitro* data on the kinetics of heterotetramer formation show that the subunit exchange occurs on a relatively slow timescale.^17^ The data on the kinetic stability of the homotetramers presented here reveal that dissociation of the kinetically more stable p73 tetramer is the rate-limiting step in the formation of mixed tetramers of p63 and p73. The higher kinetic stability of the p73 tetramer also suggests that p73 tetramers may be less prone to dominant-negative effects via formation of mixed tetramers with its isoforms than p63. However, it is clear that differential kinetic stability of the tetramers is only one factor in a complex system *in vivo*, in addition to differential expression patterns and differential control of protein degradation via ubiquitin ligases, a control mechanism recently shown for TAp73 and ΔNp73.^24^ Importantly, recent studies on different p53 variants have shown that the half-life of p53 tetramers is also modulated by domain–domain interactions not involving the oligomerization domain^25^ and interactions with accessory proteins.^26^ Future studies will show how such interactions influence the half-life of full-length p63 and p73 tetramers, as well as their numerous isoforms. Overall, the structure and stability of p53 family tetramers provide intriguing insights not only into the structural evolution of this protein family but also into the evolution of intricate molecular mechanisms regulating protein function at various stages of cell development.

## Methods

### Cloning, gene expression and protein purification

p63 and p73 tetramerization domain variants were produced as described previously.^17^ Briefly, cDNA clones of human p63 and p73 were purchased from Geneservice Ltd. Different regions of the tetramerization domain of p63 and p73 were cloned into vector pRSET-HLT using BamHI and EcoRI restriction sites. The resulting plasmid encodes a fusion protein with an N-terminal 6× His tag, followed by a lipoyl domain, a thrombin cleavage site and the p63/p73 sequence of interest. The oligomerization domains were expressed in *Escherichia coli* strain BL21 and purified using a Ni-affinity column followed by cleavage with thrombin (Sigma) overnight. Subsequent purification via a second Ni-affinity column, anion-exchange chromatography on Q Sepharose and gel filtration on Superdex 75 yielded a purity of > 99%. The purified samples were concentrated to 10–15 mg/ml, flash frozen and stored in liquid nitrogen. As a result of the cloning strategy employed, the recombinant proteins contained a Gly–Ser dipeptide at their N terminus. To produce SeMet-substituted proteins, we grew cells in M9 minimal medium supplemented with 50 mg/l SeMet and 100 mg/l essential amino acids, and the protein purification buffers were supplemented with 7 mM β-mercaptoethanol to prevent oxidation of SeMet. The p53 tetramerization domain (residues 325–356) was produced in *E. coli* Bl21 cells as untagged protein using a pRSET vector, and subsequent purification included anion-exchange chromatography on Q Sepharose and gel filtration on Superdex 75. To produce labeled p53, p63 and p73 variants for subunit exchange experiments by mass spectrometry, we grew cells in M9 minimal medium supplemented with minerals and vitamins, together with 1 g/l ^15^NH_4_Cl and ^13^C d-glucose.

### Crystallography and structure analysis

p63 oligomerization domain crystals were grown at 17 °C using the sitting-drop vapor diffusion technique. We mixed 1-μl protein solution [12–15 mg/ml protein in 20 mM Tris (pH 8.5) and 50 mM NaCl] and 1-μl crystallization buffer above a reservoir solution of 100 μl. Crystals were obtained using the following crystallization buffers: 30% polyethylene glycol 400, 0.1 M Hepes (pH 7.5) and 0.2 M Mg chloride for p63(359–388) and 10% polyethylene glycol 8000, 0.1 M Hepes (pH 7.5) and 0.2 M Ca acetate for p63(359–402) (both native and SeMet-substituted crystals). Crystals were either flash frozen directly in liquid nitrogen [p63(359–388)] or soaked in mother liquor supplemented with 20% glycerol before flash freezing [p63(359–402)]. X-ray data sets were collected on beamlines I02, I03 and I04 at the Diamond Light Source. All data were indexed and integrated using MOSFLM^27^ and further processed with SCALA.^28^ The structures were solved by multiwavelength anomalous dispersion phasing using SeMet-substituted crystals. SeMet sites were found with SHELXD^29^ using peak, inflection and remote data sets. For p63(359–388), the coordinates of these sites were imported into autoSHARP,^30^ phases were calculated and an initial model was built. For p63(359–402), phasing was performed using autoSHARP, and the initial model was built using Buccaneer.^31^ Subsequently, the models were refined using Coot,^32^ PHENIX^33^ and REFMAC5^34^ including TLS refinement (one TLS group per monomer). In the case of p63(359–402), the model was extended to higher resolution using the native data set. Data collection and refinement statistics are summarized in Table 2. The final model of p63(359–402) comprising residues 359–398 had *R*_cryst_ and *R*_free_ values of 23.5% and 28.5%, respectively. The overall *B*-factor of 81 Å^2^ reflects mobility and disorder of the molecules in the crystal, which was partly modeled through the TLS refinement. The N-terminal glycine–serine tag and the four C-terminal residues could not be modeled, which contributed to the relatively high *R*_free_ value. It is not unexpected for a structure of a small domain to have a relatively high *R*_free_ value for a given resolution because a few poorly/unmodeled disordered residues at the termini result in larger differences between calculated and measured structure factors than in larger proteins where they only marginally contribute to the total scattering. Buried surface areas were calculated using the PISA server†.^36^ Structural figures were produced using PyMOL‡.

#### Monitoring subunit exchange by mass spectrometry

In a typical subunit exchange reaction, equal amounts of [^12^C–^14^N] and [^13^C–^15^N] p53, p63 or p73 in 500 mM ammonium acetate (pH 6.9) were mixed and incubated at 37 °C. The final concentration of each component in the three exchange reactions was 10 μM (monomer concentration), that is, the measurements were performed at a concentration where all species are tetrameric. At different time points after mixing, samples were taken, and nESI-MS were recorded on a Synapt HDMS system (Waters Corporation, Milford, MA) optimized for the transmission of noncovalent complexes.^37^ We introduced 1–3 μl of the protein mixture by electrospray from gold-coated borosilicate capillaries prepared in-house as described previously.^38^ The following experimental parameters were applied: capillary voltage = 1.0–1.3 kV, sample cone = 100 V, trap and transfer collision energy = 100 V, backing pressure = 5 mbar, source pressure = 0.06–0.07 mbar, trap pressure = 0.05 mbar, ion-mobility spectrometry pressure = 0.5 mbar and time-of-flight analyzer pressure = 1.2 × 10^− 6^ mbar. Calibration, data processing, spectra simulation and kinetic modeling were performed as described previously.^39^

### Accession numbers

The atomic coordinates and structure factor amplitudes for the p63 tetramerization domain structures have been deposited in the PDB§ (PDB codes 3ZY0 and 3ZY1).

## Figures and Tables

**Table 1:** Kinetic stability of p53 family tetramers at 37 °C

Tetramer	Half-life, *t*_1/2_ (min)^a^
p53(325–356)	5 ± 0.5
p63(356–411)	7 ± 1
p73(351–399)	77 ± 5

a Determined by mass spectrometry monitoring the subunit exchange of isotopically labeled and unlabeled oligomerization domain variants.

**Table 2:** X-ray data collection and refinement statistics

Protein	p63(359–388) SeMet			p63(359–402) SeMet			p63(359–402) native
*Data collection*							
Space group	*P*1			*P*422			*P*422
*a*, *b*, *c* (Å)	30.14, 33.13, 34.68			58.21, 58.21, 39.38			58.20, 58.20, 39.33
α, β, γ (°)	105.33, 102.07, 110.15			90.00, 90.00, 90.00			90.00, 90.00, 90.00
Data set	Peak	Inflection	Remote	Peak	Inflection	Remote	Native
Wavelength (Å)	0.9796	0.9797	0.9686	0.9791	0.9793	0.9677	0.9791
Resolution (Å)^a^	31.6–1.9	31.6–1.9	31.6–1.9	58.2–2.46	58.2–2.46	58.2–2.46	58.2–2.15
	(2.0–1.9)	(2.0–1.9)	(2.0–1.9)	(2.60–2.46)	(2.60–2.46)	(2.60–2.46)	(2.27–2.15)
Unique reflections	8661	8662	8701	2700	2701	2708	3966
Completeness (%)^a^	96.5 (96.6)	96.5 (96.6)	96.3 (96.6)	99.6 (100)	99.7 (100)	99.8 (100)	99.4 (99.8)
Multiplicity^a^	3.7 (3.8)	3.7 (3.8)	3.7 (3.8)	8.5 (8.4)	8.4 (8.4)	8.5 (8.5)	9.4 (9.7)
*R*_merge_ (%)^a^^,^^b^	7.5 (9.0)	8.0 (9.1)	7.5 (9.0)	5.8 (23.7)	5.8 (23.7)	5.5 (30.0)	4.7 (80)
〈*I*/σ_*I*_〉^a^	12.1 (9.0)	12.1 (9.1)	11.5 (8.9)	21.2 (6.9)	21.2 (6.9)	21.1 (6.2)	23.7 (3.3)
Wilson *B* value (Å^2^)	20.7	20.9	20.8	66.4	67.8	69.0	52.2

*Refinement*							
Molecules per asymmetric unit	4						1
Protein/water atoms	954/50						332/0
*R*_cryst_ (%)^c^	20.4						23.5
*R*_free_ (%)^c^	25.2						28.5
RMSD bonds (Å)	0.013						0.012
RMSD angles (°)	1.5						1.4
Mean *B* value (Å^2^)	23.9						81.0
Ramachandran favored (%)^d^	100						97.4
Ramachandran outliers (%)^d^	0						0

a Values in parentheses are for the highest-resolution shell.

b *R*_merge_  = ∑(*I*_*h*__,_*_i_* − 〈*I_h_*〉)/∑*I_h_*_,*i*_

c *R*_cryst_ and *R*_free_  = ∑||*F*_obs_| − |*F*_calc_||/∑|*F*_obs_|, where *R*_free_ was calculated over 5% of the amplitudes chosen at random and not used in the refinement.

d Determined using MolProbity.^35^

## Footnotes

† http://www.ebi.ac.uk/pdbe/prot_int/pistart.html

‡ www.pymol.org

§ www.pdb.org
